# Public-private partnership models for rehabilitation service delivery: A scoping review

**DOI:** 10.4102/sajp.v79i1.1856

**Published:** 2023-05-18

**Authors:** Senzelwe M. Mazibuko, Thayananthee Nadasan, Pragashnie Govender

**Affiliations:** 1 Department of Physiotherapy, School of Health Sciences, University of KwaZulu-Natal, Durban, South Africa; 2 Senzelwisihe Rehabilitation Hospital, Empangeni, South Africa; 3 Department of Occupational Therapy, University of KwaZulu-Natal, Durban, South Africa

**Keywords:** public-private mix, public-private cooperation, public-private coordination, public-private collaboration, physical therapy, physiotherapy, occupational therapy, speech therapy, evidence synthesis

## Abstract

**Background:**

Public-private partnership (PPP) for the delivery of health services is known to improve access to healthcare, yet little is known about its utilisation for rehabilitation services, particularly in sub-Saharan Africa (SSA).

**Objectives:**

As a first step to generating evidence to develop a PPP model for physiotherapy service delivery in South Africa, our study mapped and described available research evidence on PPP models for rehabilitation services in the global literature.

**Method:**

The Arksey and O’Malley framework guided our scoping review. Published research on rehabilitation and PPP was searched in five databases from 2000 to August 2022 using keywords, Medical Subject Headings (MeSH) and Boolean terms. Two reviewers independently completed the titles, abstracts and full-text screening of the articles and data extraction from the included articles. A narrative synthesis was conducted, and summaries of the findings are reported.

**Results:**

Nine articles were included from a total of 137 obtained from the evidence searches. Of these, five were from Australia and the others from Hong Kong, Denmark, Bangladesh and the Netherlands. All the included articles showed evidence of PPP models for physiotherapy service delivery.

**Conclusion:**

Our study suggests that PPP models for physiotherapy service delivery exist, particularly in high-income countries (HICs). It also highlights limited research in low- and middle-income countries (LMICs).

**Clinical implications:**

There is a need for primary studies to generate further evidence and develop innovative PPP models for rehabilitation services for the populations who need them most as part of efforts towards improving access to healthcare in LMICs.

## Background

Disability is a global health issue. An estimated 1.3 billion people, or roughly 16% of the global population, are disabled (World Health Organization 2022). This figure rises as the population ages and the prevalence of non-communicable diseases rises (World Health Organization 2022). In high-income countries (HICs), the estimated prevalence of disability is reported to be about 12% compared to 18% in low- and middle-income countries (LMICs) (World Health Organization 2011). Persons with disabilities (PLWDs) are a diverse group. Factors such as sex, age, gender identity, sexual orientation, religion, race, ethnicity and economic situation affect their life experiences and health needs (World Health Organization 2022). Persons with disabilities die earlier, have poorer health and experience more limitations in everyday functioning than others (World Health Organization 2022). Studies have shown that the prevalence of disability and poverty has a direct relationship (the more disabilities, the more poverty), exacerbated in rural areas by inadequate or non-existent healthcare services (Dayal 2010; Mji et al. 2013; M’Kumbuzi & Myezwa 2016; Sherry 2014).

Persons with disabilities have a right to quality healthcare as outlined by the United Nations Convention on the Rights of Persons with Disabilities (UNCRPD). However, health services for PLWDs are limited due to limited human resources, dilapidated infrastructure, overburdened public health systems and limited financial resources (Hanass-Hancock et al. 2017; Sherry 2014; Visagie & Swartz 2016). People living with disability, for example, have limited access to rehabilitation services in sub-Saharan Africa (SSA) (Naidoo & Ennion 2019). This has been attributed to a lack of funding for the construction of rehabilitation centres and the high financial costs associated with rehabilitation (Naidoo & Ennion 2019), among others, such as transportation costs to the facility, distance and poor knowledge by PLWDs of where these services are, or knowledge about rehabilitation. Furthermore, referral pathways are erratic, and the availability of rehabilitation services is compromised, resulting in avoidable complications for patients due to inadequate follow-up (Health 2015; Naidoo & Ennion 2019; Sherry 2014; Visagie, Scheffler & Schneider 2013; Visagie & Swartz 2016). The rehabilitation challenges are exacerbated by a lack of infrastructure, particularly in SSA, where district hospital rehabilitation units are poorly maintained and scarce (Health 2015). There has been little to no research on appropriate rehabilitation development indicators at a tertiary, specialised or primaryhealthcare (PHC) levels (Health 2015). Currently, communitieswith limited resources rely on community-based rehabilitation (CBR), friends, family and other community groups (World Health Organization 2011).

A public-private partnership (PPP) model has been identified as a critical strategy for improving public health systems and mitigating the rising costs of a private healthcare sector that is already expensive and unsustainable (Myezwa & Van Niekerk 2013; Raman & Björkman 2015). A PPP is an agreement between a government institution and a private party in which (1) the private party performs an institutional function and/or uses state property in terms of output specifications, and (2) substantial project risk (financial, technical, operational) is transferred to the private party, with the private party benefiting from unitary payments from government budgets and/or user fees (Manuel 2007).

There has been an increasing interest in implementing PPPs in some SSA countries to improve public health systems (Kula & Fryatt 2014; Loxley 2013; Mabunda, London & Pienaar 2018; Thadani 2014; Walwyn & Nkolele 2018). In Lesotho, for instance, the private sector helped to renovate and redevelop Queen Mamohato Memorial Hospital in Maseru, a public institution (Lang 2016). Moreover, the government collaborated with a local church in Uganda to build Ruharo Mission Hospital (Asasira & Ahimbisibwe 2018). Consequently, PPPs can leverage resources by forming mutually beneficial partnerships with private healthcare providers to create an effective, efficient and responsive public health sector by transferring private sector technical skills, innovation and resources (Raman & Björkman 2015; Suchman, Hart & Montagu 2018; Whyle & Olivier 2016). Globally, governments are tasked with improving their healthcare systems to meet the needs of most citizens who rely on public health systems. As a first step towards generating evidence to develop a PPP model for physiotherapy services in South Africa, our study systematically mapped and described available research evidence on PPP models for rehabilitation service delivery in a global context.

## Method

Our scoping review followed the five steps outlined by Arksey and O’Malley in their methodological framework – identifying the research question; identifying relevant studies; study selection; charting the data; and collating, summarising and reporting results (Arksey & O’Malley 2005).

### Identifying research question(s)

Our review answered the following question: ‘What research evidence exists on PPP models for rehabilitation services delivery in the global context?’ The population, concept and context for our study’s question are defined in Box 1.

BOX 1 Population, concept and context framework for the main review question.

**Table:** 

Population	Patients of all ages who use rehabilitation services such as occupational therapy, physiotherapy, speech therapy and audiology.
Concept	Public-private partnership: This refers to a contract between a private party and a government agency for providing a public service in which the private party bears significant risk and management responsibility (Forrer et al. 2010).
Context	Global

### Identifying relevant studies

Relevant peer-reviewed articles and grey literature were sourced from PubMed, EBSCOhost (Academic search complete, CINAHL with full text, Health Sources), Cochrane Library, SCOPUS and Google Scholar from 01 January 2000 to 12 August 2022. In consultation with an expert librarian, a search strategy was developed using keywords (‘medical rehabilitation’, ‘physical therapy’, ‘physiotherapy’, ‘occupational therapy’, ‘speech therapy’, ‘public-private partnership’, ‘public-private mix’, ‘public-private cooperation’, ‘public-private coordination’, ‘public-private collaboration’, ‘contract out’, ‘contracting out’, ‘Private finance initiative contracts’), Boolean terms (and/or) and Medical Subject Heading (MeSH) terms. The first author (S.M.M.), a physiotherapist, conducted the literature search with support from an expert librarian. EndNote X 20 was used to manage all citations.

## Eligibility criteria

### Inclusion criteria

- Peer-reviewed publications.
- Grey literature.
- Any language.
- Publication between 2000 and August 2022.
- Articles that focused on rehabilitation services delivery (occupational, physiotherapy, speech therapy and audiology, psychologists, social workers and dieticians and/or nutritionist).
- Articles that involved PPP for rehabilitation service delivery.
- Articles presenting evidence on access to rehabilitation services.
- Articles presenting evidence on referral pathways in rehabilitation.
- Articles presenting PPP models and/or frameworks for rehabilitation service delivery.
- Primary study designs.
- Frameworks or models.

### Exclusion criteria

- Studies that focused on PPP policies, other healthcare services such as for human immunodeficiency virus (HIV), malaria, tuberculosis and others.
- Studies that had no link with the PPP policies.
- Articles published before 2000.

### Study selection

The EndNote library was cleaned by identifying and removing all duplicate articles and shared with the review team. The screening tools were piloted by two reviewers independently using 10% of the articles. The necessary adjustments based on the feedback received were made to ensure the screening tools were accurate and reliable. Based on the eligibility criteria, two reviewers independently screened and categorised the articles into either the ‘include’ or ‘exclude’ group at the titles and abstracts and the full-text articles screening stages. A third reviewer resolved differences in the responses of the two independent reviewers at both screening stages. The University of KwaZulu-Natal library service was used to retrieve full-text articles that were not open-access publications for screening.

### Charting data

A data extraction form was developed to chart relevant data from the included articles. Two reviewers independently extracted data from the included studies using a pilot-tested form. A third reviewer was employed to resolve any discrepancies. To answer our study’s question, we extracted the following relevant data: author information and publication year, study objective or aim, study setting or location (country), type of rehabilitation service delivery (service domain), services provided, type of model and targeted population.

### Collating, summarising and reporting the results

A narrative synthesis summarised all relevant data to answer our study question. A flowchart diagram (Page et al. 2021) was used to present the results of our study selection, while a table presents the characteristics of the included studies and results. In addition, a narrative summary of our findings is presented.

## Results

### Study selection

**FIGURE 1:**
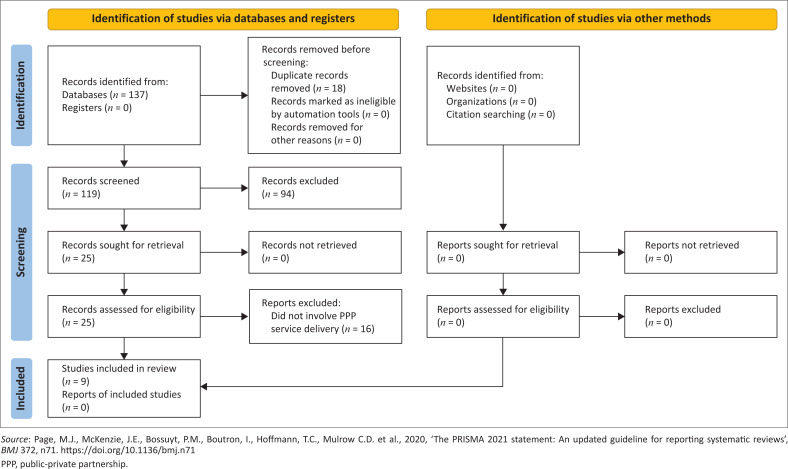
PRISMA 2020 flow diagram.

A total of 137 articles was obtained from the database searches. Of the 137 articles, 18 duplicates were identified and removed. Subsequently, 119 articles were screened, and 110 which did not meet our inclusion criteria during the titles, abstracts and full-text screening phases were excluded from the data extraction and synthesis (Figure 1).

### Characteristics of the included studies

Of the nine included studies, approximately 56% (*n* = 5) were from Australia (Farquhar, Moran & Schmidt 2020; Schmidt & Dmytryk 2014) and the remainder 44% (*n* = 4) from Hong Kong (Schoeb 2016), Denmark (Larsen, Aust & Høgelund 2017), Bangladesh (Al Imam et al. 2022) and the Netherlands (Ijntema et al. 2022) with a study each. Qualitative study designs were mostly retrieved (44%). All included studies focused on physiotherapy services. The PPP models discussed by these included studies were as follows: business model (Al Imam et al. 2022; Carr, Kidd & Maloney 2013; Farquhar et al. 2020; Ijntema et al. 2022), informal PPP recruitment model (Schmidt & Dmytryk 2014), new graduate physiotherapists’ education PPP model (Schmidt & Dmytryk 2017), individual funding (IF) model (Dew et al. 2013), social health model (Schoeb 2016) and Falck’s rehabilitation model (Larsen et al. 2017). The services provided varied and primarily targeted all individuals requiring physiotherapy (Table 1).

**TABLE 1:** Characteristics of included studies.

Author (year)	Location/setting	Objective(s)	Type of study	Type of medical rehabilitation service	Services provided	Model type	Target group
Carr et al. (2013)	Australia	To test the viability of a business model that utilised three different funding streams (local health district, Medicare local and private) to establish a physiotherapy service to rural communities in south-western New South Wales.	Pilot study	Physiotherapy	A private physiotherapist from a regional township provided outreach physiotherapy services to two rural communities where service gaps had been identified. Use of allied health assistants to enhance the service between visits from the physiotherapist. Shared governance arrangements established between the local health district and the private physiotherapist to train and supervise allied health assistants.	Business model	Acute and postacute clients of the local health district, patients with chronic disease referred under a GP management plan, and privately funded clientele.
Farquhar et al. (2020)	Australia	To investigate how success of a PPP model of service delivery in a rural setting was defined from the perspective of stakeholders from each organisation as well as identify the barriers and enablers.	Qualitative (constructive inquiry design)	Physiotherapy	Private physiotherapy businesses supplied physiotherapy services to hospital inpatients, aged care facility residents and outpatients in four outer regional Australian towns.	Business public-private physiotherapy service delivery model	All individuals
Schmidt and Dmytryk (2014)	Australia	To examine the value and potential of the PPP model in recruiting and managing new graduate physiotherapists.	Qualitative (case study using appreciative enquiry)	Physiotherapy	Shared recruitment, orientation, management and ongoing education of new graduate physiotherapists.	Informal PPP recruitment model	New graduate physiotherapists
Schmidt and Dmytryk (2017)	Australia	To explore the shared education process and the value of learning experiences for each sector (public and private).	Qualitative study (appreciative enquiry)	Physiotherapy	An informal PPP created a local public hospital and local private physiotherapists for the purposes of attracting and educating new graduate staff.	New graduate physiotherapists education PPP model	New graduate physiotherapists
Dew et al. (2013)	Australia	To describe some benefits and barriers to using individual funding to access therapy services in rural areas.	Qualitative study	Physiotherapy	Introduction of a national disability insurance scheme to provide individual funding to nongovernment organisations to deliver specialist services such as accommodation, therapy services, day programmes or employment to children with disability	Individual funding model	Children with disability
Schoeb (2016)	Hong Kong	To analyse the challenges facing Hong Kong in view of the social model of health.	Review	Physiotherapy	Use of the Elderly Health Care Voucher Scheme vouchers for services provided by private practitioners, including physiotherapists.	Social health model	Older people (over 70 years)
Larsen et al. (2017)	Denmark	To investigate whether a multidimensional PPP intervention, focussing on improving the quality and efficiency of sickness benefit case management, reduced the sickness benefit duration and the duration until self-support.	Quasi-experimental study (difference-in-difference design)	Physiotherapy	Implementing Falck’s rehabilitation model with integrated case management, job coaching and healthcare services, with diagnoses and treatment by physiotherapists and others. Social insurance officers, the job coach and medical expert were located in the same building where they cooperate to establish return-to-work interventions that better integrate medical and employment-oriented aspects.	Falck’s rehabilitation model	All individuals
Al Imam et al. (2022)	Bangladesh	To report the lesson learnt in establishing a social business model of early intervention and rehabilitation services for children with CP and adults with disabilities in a rural sub-district.	Case study	Rehabilitation services	Provided early intervention and rehabilitation services	Social business model	Children with CP and adults with disabilities in a rural sub-district
Ijntema et al. (2022)	the Netherlands	To delineate the relations between business model efficiency and novelty and PTPHO-centred outcomes while accounting for managed competition contracts in Dutch healthcare.	Quantitative (cross-sectional design)	Physiotherapy	Introduction of business principles to foster efficient healthcare by way of managed competition (a contract between a health insurer and a physiotherapy primary healthcare organisation).	Business model	All individuals

GP, general practitioner; PPP, public-private partnership; CP, cerebral palsy; PTPHO, physiotherapy primary healthcare organisation.

## Study findings

### Business models

Of the nine included articles in the review, four from Australia reported research findings that involved a business model (Carr et al. 2013; Farquhar et al. 2020), Bangladesh (Al Imam et al. 2022), and the Netherlands (Ijntema et al. 2022).

In Australia, Carr et al. investigated the feasibility of a business model that used three different funding sources (local health district, Medicare local, and private) to establish a physiotherapy service in rural communities in south-western New South Wales (Carr et al. 2013). The pilot initial results showed a high level of uptake for previously unavailable services in primary care, residential aged care and acute and/or subacute care across a wide geographical area (Carr et al. 2013). Oversubscription of available services in some communities in the latter stages of the pilot was also reported (Carr et al. 2013). Farquhar et al. investigated how the success of a business public-private physiotherapy service delivery model in a rural setting was defined by stakeholders from each organisation and identified the barriers and enablers (Farquhar et al. 2020). All participants deemed the model successful (Farquhar et al. 2020). Critical success factors were vital to improved access to local services and satisfied stakeholders (Farquhar et al. 2020). Three mechanisms were identified to implement the service delivery model (Farquhar et al. 2020). The first mechanism was the provision of human and other resources, which included the workforce model and the use of various partnership resources (Farquhar et al. 2020). The second mechanism was stakeholder engagement, which required both motivated and consistent stakeholders (Farquhar et al. 2020). The third mechanism was streamlined processes, which included the contract and referral schedule content, streamlined administration processes for contracting and accounting, processes for managing private therapists in a public setting and communication processes (Farquhar et al. 2020).

In Bangladesh, Al Imam et al. (2022) investigated the lessons learned in establishing a social business model (model centre cost ~$5955.00 with an average monthly running cost of ~$994.00) of early intervention and rehabilitation services for children with cerebral palsy (CP) and adults with disabilities in a rural sub-district (Al Imam et al. 2022). During the 17-month study period, 862 patients with musculoskeletal and neurological disorders received 7038 therapy sessions (an average of eight sessions per patient) (Al Imam et al. 2022). Low back pain was the most common clinical presentation (35.6%; *n* = 307). Six percent (*n* = 52) of the attendees were children with CP (mean [standard deviation] age: 6.3 [4.0] years; 35.7% [*n* = 19] were female), and they received 1392 sessions, with each child receiving an average of 27 sessions (Al Imam et al. 2022). The centre broke even in the 13th month and remained profitable for the remaining 4 months of the study period (Al Imam et al. 2022). In 2018 and 2019, an average session fee of $2.20 resulted in a gross margin of −$1458.00 and $1940.00, respectively (Al Imam et al. 2022). Revenue-to-cost ratios were 0.27:1 and 0.51:1, respectively, with average rates of return of 41.4% and 10.1% (Al Imam et al. 2022). Sensitivity analysis revealed that at session fees of $3.00, $2.50, $2.00, $2.00, $1.50 and $1.50, session numbers of 5000, 6000, 7000, 8000, 9000 and 10 000, respectively, were required to break even (Al Imam et al. 2022).

In the Netherlands, Ijntema et al. (2022) delineated the relationships between business model efficiency and novelty and physiotherapy primary healthcare organisation (PTPHO)-centred outcomes in Dutch healthcare while accounting for managed competition contracts (Ijntema et al. 2022). The study showed that managed competition and business model efficiency do not affect PTPHO outcomes (Ijntema et al. 2022). The managed competition contract does not appear to moderate the business model efficiency and PTPHO-centred outcomes relationship (Ijntema et al. 2022). Business model novelty correlates positively with PTPHO-centred outcomes (Ijntema et al. 2022). A managed competition contract is discovered to moderate the relationship between business model novelty and PTPHO-centred outcomes (Ijntema et al. 2022).

### New graduate physiotherapists training public-private partnership model

In Australia, Schmidt and Dmytryk investigated the shared education process and the value of learning opportunities in each sector (public and private) (Schmidt & Dmytryk 2017). The study showed that the model offered a novel approach to providing comprehensive new graduate physiotherapy education in a rural setting (Schmidt & Dmytryk 2017). Although the study only included two small organisations, the experience demonstrated that the public and private sectors working together to provide education leveraged the strengths of both sectors (Schmidt & Dmytryk 2017).

### Informal public-private partnership model for recruitment and management of new graduate physiotherapists

In Australia, Schmidt and Dmytryk studied the value and potential of the PPP model in recruiting and managing new graduate physiotherapists (Schmidt & Dmytryk 2014). The findings revealed that the ability to attract high-quality applicants to difficult-to-fill positions reduced the risk of new-graduate attrition due to social isolation and enhanced networking between sectors, and enhanced staff skill development through diverse clinical and nonclinical experiences. These were all organisational benefits of a shared public-private role (Schmidt & Dmytryk 2014). The model was based on management flexibility and had the potential to be extended to other areas and professions (Schmidt & Dmytryk 2014). Dedicated funding, targeted recruitment strategies and increased planning to ease the transition into the workplace were identified as potential facilitators to improve the model even further (Schmidt & Dmytryk 2014).

### Individual funding model

In Australia, the study by Dew et al. described the benefits and barriers to using IF to access physiotherapy services in rural areas (Dew et al. 2013). Individual funding improved access to and choice of physiotherapy providers (Dew et al. 2013). The barriers identified were the lack of information and advice, limited local service options and capacity, higher costs and fewer services, and complexity of self-managing packages (Dew et al. 2013). Carers expressed a desire for easily accessible information, a local point of contact for support and guidance, adequate financial compensation to offset additional travel expenses, and coordinated eligibility and accountability systems to address the barriers to using IF in rural and remote areas (Dew et al. 2013). At the same time, service providers needed coordinated cross-sector approaches, local workforce planning to address therapist shortages, certainty about service viability and growth, and transparent policies and procedures for implementing IF to address barriers to using IF in rural and remote areas (Dew et al. 2013).

### Falck’s rehabilitation model

In Denmark, Larsen et al. investigated whether a multifaceted PPP intervention aimed at improving the quality and efficiency of sickness benefit case management reduced sickness benefit duration and time to self-sufficiency (Larsen et al. 2017). There was no joint effect of the intervention on the duration of sickness benefits (hazard ratios [HR]: 1.02, confidence interval [CI]: 0.97–1.07) or the duration until self-sufficiency (HR: 0.99, CI: 0.96–1.02) (Larsen et al. 2017). The effect varied by municipality, with sickness benefit HRs ranging from 0.96 (CI: 0.93–1.00) to 1.13 (CI: 1.08–1.18) and self-support HRs ranging from 0.91 (CI: 0.82–1.00) to 1.11 (CI: 1.08–1.18). (CI: 1.06–1.17) (Larsen et al. 2017).

### Social health model

In Hong Kong, Schoeb’s study examined the challenges confronting Hong Kong in light of the health social model (Schoeb 2016). The Hong Kong Special Administrative Region combines a British colonial history with a Chinese cultural context. It provides its residents with a dual system that includes a comprehensive and efficient public healthcare system and private hospitals and practitioners (Schoeb 2016). Due to an ageing population, staff shortages at all levels, an underdeveloped PHC system and increasing demand for health services are all looming challenges (Schoeb 2016).

## Discussion

Our study mapped and described research evidence on PPP models for rehabilitation services in the global literature. The results showed a variety of PPP models, such as business (Al Imam et al. 2022; Carr et al. 2013; Farquhar et al. 2020; Ijntema et al. 2022), recruitment (Schmidt & Dmytryk 2014), new graduate training (Schmidt & Dmytryk 2017) and IF (Dew et al. 2013), exist for physiotherapy service delivery. The PPP arrangement facilitated access to physiotherapy services, especially in rural areas. However, most PPP models were developed and implemented in HICs, with a few in LMICs.

Like HICs, the private sector plays an essential role in healthcare services delivery, including rehabilitation services in most LMICs (Bhattacharyya et al. 2010), partly because the public health system is unable to provide the needed health services to all who need them (Bhattacharyya et al. 2010). Aside from infrastructure challenges for rehabilitation in most LMICs, there is also a problem with human resource capacity for public hospitals or clinics, such as inadequate medical rehabilitation professionals (Fusheini & Eyles 2016). Governments, particularly in low-income countries, struggle to retain the few trained rehabilitation professionals partly due to remuneration concerns (Rockers et al. 2012). Nonetheless, the United Nations advocates universal health coverage as stipulated by sustainable development goal 3.8. All member countries are urged to achieve this goal by 2030 (United Nations 2016). Therefore, we encourage governments in LMICs to partner with private rehabilitation providers to expand access to PLWDs, particularly those who are poor and cannot afford the services from private providers that are not available in public facilities. Most often, it is argued that private health services are generally expensive. Still, it is our opinion that through a PPP arrangement acceptable to all key stakeholders, their services may be affordable to even the less privileged person living with a disability requiring rehabilitation.

Although our study identified several PPP models for physiotherapy service delivery, their implementation and sustainability may be challenging in some settings, considering the economic differences among countries. We believe that further research, such as primary studies or implementation research, would be required to address contextual issues before their adoption and full implementation. Moreover, such studies may help generate further evidence to develop an innovative PPP model(s) for various rehabilitation service delivery relevant to context and potential to address service gaps in public hospitals or clinics.

Although our study has several strengths, including the inclusion of global literature, it may have inherent limitations. Few (five) databases were searched. Aside from that, our study was focused on only PPP models. Despite these limitations, our study is novel. It could provoke more research in this area, especially PPP arrangements for other rehabilitation services such as occupational therapy, speech and audiology.

In conclusion, our findings indicate that PPP models for physiotherapy service delivery exist, mainly in HICs. It also emphasises the scarcity of research on PPP models and rehabilitation services in LMICs. To improve access to healthcare in LMICs, we recommend primary studies to generate additional evidence and develop innovative PPP models for rehabilitation services for the populations who need them the most.
